# RETRACTION: Nanoscale Modification of Titanium Implants Improves Behaviors of Bone Mesenchymal Stem Cells and Osteogenesis In Vivo

**DOI:** 10.1155/omcl/9831451

**Published:** 2025-07-08

**Authors:** Oxidative Medicine and Cellular Longevity

RETRACTION: H. Li, J. Huang, Y. Wang, Z. Chen, et al., “Nanoscale Modification of Titanium Implants Improves Behaviors of Bone Mesenchymal Stem Cells and Osteogenesis In Vivo,” *Oxidative Medicine and Cellular Longevity*, 2022, no. 1 (2022): 1–13. https://doi.org/10.1155/2022/2235335.

The above article, published online on 4 January 2022 in Wiley Online Library (wileyonlinelibrary.com), has been retracted following the agreement of the journal's Chief Editor Jeannette Vasquez Vivar; and John Wiley & Sons Ltd.

The retraction has been agreed following an investigation undertaken by the publisher, which identified concerns related to the authenticity of Figure 5e, in which multiple areas were found to be unexpectedly duplicated.

As a result of the investigation, the data of this article are considered unreliable.

The authors had been informed of this retraction but did not provide a response.

